# Protective effects of gypenosides on LDL-induced myocardial injury through the miR-223/NLRP3 axis in hyperlipidemia

**DOI:** 10.3389/fnut.2026.1740653

**Published:** 2026-04-01

**Authors:** Wei Peng, Qiong Zhang, Tingjuan Wang, Zhijie Wang, Menghui Zhao, Yue Li, Xiaolin Chen, Yan Zhang, Jie Yang, Linsheng Huang, Xiang Liu, Feifeng Li

**Affiliations:** 1 Health Management Center, Shiyan Renmin Hospital, Hubei University of Medicine, Shiyan, China; 2 Department of Preventive Medicine, School of Public Health, Hubei University of Medicine, Shiyan, China; 3 Hubei Key Laboratory of Wudang Local Chinese Medicine Research, Hubei University of Medicine, Shiyan, China; 4 Department of Drug Quality Inspection, School of Pharmaceutical Sciences, Hubei University of Medicine, Shiyan, China; 5 Department of Hepatobiliary Pancreatic Surgery, Taihe Hospital, Hubei University of Medicine, Shiyan, China

**Keywords:** gypenosides, hyperlipidemia, LDL, miR-223, NLRP3

## Abstract

**Introduction:**

Elevated low-density lipoprotein cholesterol (LDL-C) is a major contributor to hyperlipidemia and cardiovascular risk. This study examined the association of LDL-C with metabolic abnormalities in a health examination population and investigated whether gypenosides (GPs) protect against LDL-induced cardiomyocyte injury through the miR-223/NLRP3 axis.

**Methods:**

A cross-sectional analysis was conducted in 19,862 adults undergoing routine health examinations. In parallel, H9C2 cardiomyocytes were exposed to LDL with or without GPs. Cell viability, cell cycle distribution, wound healing, Matrigel-based tube formation, oxidative stress, inflammatory markers, and miR-223/NLRP3-related signaling molecules were assessed. A miR-223 inhibitor and the NLRP3 inhibitor MCC950 were used to further examine the mechanism.

**Results:**

Elevated LDL-C was more common in middle-aged and older men and was associated with higher blood glucose, white blood cell count, body mass index, systolic blood pressure, total cholesterol, and triglycerides, together with lower HDL-C. In H9C2 cells, LDL induced abnormal proliferative activation and cell-cycle disturbance, reduced wound healing and tube-like network formation, increased ROS, NO, and LDH release, activated the NLRP3/NF-κB/p38/IL-6 pathway, and suppressed miR-223 expression. GPs attenuated these changes, restored miR-223 expression, and reduced inflammatory signaling. Inhibition of miR-223 weakened the protective effects of GPs, whereas MCC950 suppressed LDL-induced inflammatory activation.

**Conclusion:**

Elevated LDL-C was associated with metabolic and inflammatory disturbances, and LDL directly induced cardiomyocyte injury *in vitro*. More importantly, Gypenosides markedly alleviated LDL-induced cellular injury by restoring miR-223 expression and suppressing NLRP3-mediated inflammatory signaling. The inhibitory effect of MCC950 further supports a central role for NLRP3 in this process. Together, these findings suggest that Gypenosides may have therapeutic potential for hyperlipidemia-related myocardial injury.

## 1 Introduction

Cardiovascular diseases (CVDs) claim approximately 17 million lives annually worldwide (1, 2), with hyperlipidemia constituting the primary modifiable risk factor for cardiovascular morbidity and mortality (3). The pathological significance of hyperlipidemia lies in its direct cardiotoxic effects, which induce structural remodeling and functional impairment of cardiomyocytes (4). Chronic lipid overload promotes myocardial lipid deposition while initiating systemic oxidative stress and inflammatory cascades, which are key drivers of progressive myocardial dysfunction and fatal cardiovascular events (4). Among various lipid components, low-density lipoprotein (LDL) and its derivatives, particularly oxidized LDL (ox-LDL) and LDL-cholesterol (LDL-C), have emerged as principal mediators of hyperlipidemia-induced myocardial injury (5). Mechanistically, ox-LDL activates the NLRP3 inflammasome pathway, triggering pro-inflammatory cytokine release (5, 6), while native LDL exacerbates myocardial inflammatory stress responses (7, 8). These findings underscore the critical need to elucidate the mechanisms underlying hyperlipidemia-mediated cardiotoxicity.

Despite LDL-C being the primary therapeutic target for hyperlipidemia and its complication management, current clinical control rates remain alarmingly suboptimal (9). Recent epidemiological data indicate that 74.5% of high-risk and 93.2% of very high-risk patients fail to achieve their target LDL-C levels (9, 10). Compounding this issue is the escalating prevalence of hyperlipidemia and myocardial injury among younger populations and urban communities (11, 12), driven by rapid urbanization, dietary pattern shifts, and sedentary lifestyles (9, 13, 14). Beyond its established role in atherogenesis, emerging evidence suggests that hyperlipidemia directly impairs cardiac function through multiple pathways: enhancing susceptibility to ischemia/reperfusion injury, attenuating endogenous cardioprotective mechanisms (e.g., ischemic preconditioning), and disrupting myocardial energy metabolism (15). This dual vascular-cardiac pathogenicity highlights the urgency for further mechanistic investigations.

While LDL-C serves as the clinical gold standard for lipid monitoring, its biochemical complexity extends beyond simple cholesterol quantification (16). As a dynamic lipoprotein particle containing apolipoproteins, phospholipids, and cholesteryl esters, LDL exhibits multifaceted biological activities in lipid metabolism and cardiovascular pathophysiology (16). To address this knowledge gap, we used an *in vitro* cardiomyocyte model to systematically investigate mechanisms of LDL-induced myocardial cellular injury.

The emerging regulatory role of microRNAs (miRNAs) in lipid metabolism and inflammation has opened new therapeutic frontiers (17–19). Particularly noteworthy is miR-223-3p, which demonstrates dual cardioprotective functions: attenuating ox-LDL-induced inflammation through NLRP3 inflammasome inhibition (5) and suppressing NF-κB-mediated pro-inflammatory cytokine production (20). However, its precise role in hyperlipidemia-associated cardiotoxicity remains undefined. This knowledge gap motivated our investigation into gypenosides (GPs), phytochemicals derived from *Gynostemma pentaphyllum* with demonstrated anti-inflammatory and lipid-modulating properties (21, 22). GPs exhibit multimodal cardioprotective effects through NF-κB/NLRP3 pathway modulation (23), cholesterol efflux enhancement, and mitochondrial function optimization (24) positioning them as promising therapeutic candidates for hyperlipidemia-related cardiac dysfunction.

In this study, the population-based analysis revealed gender-specific dyslipidemia patterns, with middle-aged and elderly males exhibiting the strongest correlations between LDL-C and glycemic parameters, leukocytosis, and lipid profile abnormalities. Subsequent *in vitro* experiments demonstrated LDL's dual effects on cardiomyocytes: a paradoxical acceleration of proliferation coupled with cell cycle dysregulation, impaired migration capacity, and angiogenic-like behavior. GPs administration effectively mitigated LDL-induced cellular damage through a novel miR-223/NLRP3 regulatory axis. Mechanistically, GPs upregulated miR-223 expression to suppress NLRP3 inflammasome activation, thereby inhibiting downstream NF-κB/MAPK signaling and IL-6 overproduction. Pharmacological validation using miR-223 inhibitors and the NLRP3-specific antagonist MCC950 confirmed both the central role of NLRP3 in LDL-induced inflammation and miR-223 as the primary therapeutic target of GPs in LDL-induced myocardial injury. These findings provide a mechanistic foundation for developing miRNA-targeted therapies and phytochemical-based interventions for cardiovascular disorders related to lipid metabolism.

## 2 Materials and methods

### 2.1 Study population and data collection

The study cohort comprised 19,862 adults undergoing routine health examinations at Taihe Hospital and Renmin Hospital (Shiyan, China), representing employees of a state-owned enterprise. Standardized protocols included electrocardiographic assessment; venous blood collection for biochemical profiling; anthropometric measurements (weight, height, blood pressure, age, and sex); laboratory quantification of fasting blood glucose; lipid panel (LDL-C, HDL-C, total cholesterol, triglycerides); and complete blood counts (WBCs).

The epidemiological dataset used in this study consists of de-identified health examination records previously collected within our research group under an approved protocol. The original data collection was reviewed and approved by the Ethics Committee of Hubei University of Medicine, Reference number 2022-RE-039. For the present analysis, only anonymized data were accessed. Therefore, informed consent requirements were determined by the approving committee. LDL-C quantification was performed using automated analyzers with strict quality control measures. The study cohort consisted of 16,773 males and 3,089 females, with an age range of 18–88 years and a median age of 31 ± 12 years. Inclusion criteria for hyperlipidemia required meeting at least one of the following: LDL-C ≥ 3.4 mmol/L, total cholesterol ≥ 5.2 mmol/L, triglycerides ≥ 1.7 mmol/L, or HDL-C ≤ 1.0 mmol/L (males) or ≤ 1.3 mmol/L (females). Exclusion criteria included individuals receiving lipid-lowering therapies; pregnancy or lactation status; heavy alcohol consumption (>40 g/day); active smoking (>10 cigarettes/day); and a history of CVDs, genetic disorders, or chronic systemic diseases.

### 2.2 Epidemiological analysis

Data analysis was conducted using SPSS 25.0 (IBM Corp., Armonk, NY, United States) with triple verification protocols. Descriptive statistics were calculated (mean ± SDs and frequency distributions). Pearson correlation analysis was used to evaluate relationships among continuous variables (BMI, blood pressure, glucose, WBCs, and LDL-C). Multiple regression analysis was performed to assess the effects of total cholesterol, BMI, blood glucose, and other variables on LDL-C, and standardized regression coefficients (Beta with 95% confidence intervals) were calculated. Chi-square tests were used to analyze the associations between categorical variables (sex, hypertension status) and LDL-C.

### 2.3 Cell culture and experimental design

H9C2 cardiomyocytes, which were generously provided by Ma et al. (25) and Yan et al. (26), were maintained in DMEM supplemented with 10% FBS and 1% penicillin/streptomycin in a 37 °C/5% CO_2_ incubator. Cells in the logarithmic growth phase were subjected to serum starvation for 12 h (2% serum) prior to treatment. The experimental groups were divided (*n* = 6 per condition) as follows: the control group (NC) was treated with DMEM; the GPs group was treated with DMEM containing 500 μg/ml of GPs (Solarbio, Beijing, China); the LDL group was treated with DMEM containing different concentrations of LDL (0.25, 1.3, and 3 mg/ml); and the LDL+GPs group was treated with DMEM containing 3 mg/ml of LDL and 500 μg/ml of GPs. In mechanistic experiments, cells were cultured to 60%−70% confluence, after which LDL or GPs were added to each group. The cells were then transfected with a miR-223 inhibitor or a negative control inhibitor using GP-RNA-MATE (GenePharma, G04005, Shanghai, China) in serum-free DME. After 30 min, the medium was replaced with DMEM containing 2% FBS, and the transfection complexes were added to achieve a final concentration of 50 nM. The cells were cultured for an additional 36 h. In some experiments, cells were pretreated with MCC950 (MCE, Cat# HY-12815, United States) at a final concentration of 10 μM for 2 h, followed by replacement with 2% FBS-containing medium and the addition of LDL or GPs, and then cultured for a further 36 h.

### 2.4 Preparation and application of native LDL

Native human low-density lipoprotein (LDL) was purchased from Sigma-Aldrich (St. Louis, MO, United States; SAE0053). According to the manufacturer's specifications, the LDL preparation was isolated from human plasma and was not subjected to experimental oxidation, acetylation, or other chemical modifications. The reagent was stored at −80 °C prior to use, protected from light during handling, and freshly prepared for each experiment. Based on previously reported clinically relevant plasma LDL concentration ranges (27), LDL at 0.25, 1.3, and 3 mg/ml was applied to H9C2 cardiomyocytes to establish graded exposure conditions and assess the dose–response relationship. These concentrations correspond to levels below guideline-recommended LDL-C treatment targets, within the upper range of clinically observed LDL-C levels, and to supraphysiological conditions modeling severe hyperlipidemic stress, respectively. The highest LDL concentration (3 mg/ml) was used solely to model pathophysiological lipid overload and to induce reproducible cellular stress responses in this simplified *in vitro* system; it was not intended to represent physiological LDL exposure.

### 2.5 Cell viability and cytotoxicity assessment

The CCK-8 kit (Biosharp, Hefei, China) was used to assess cell viability. Cells were seeded in 96-well plates and treated under various conditions, including NC, GPs, LDL, and combined treatment groups. After treatment, cells were incubated with 10% (v/v) CCK-8 reagent (Biosharp) for 1 h. Absorbance at 450 nm was measured using a microplate reader (Beckman, Fullerton, CA, United States).

Lactate dehydrogenase (LDH) release was measured using an LDH kit (Beyotime, Shanghai, China). Cells were seeded and treated as described above. Supernatants were collected, centrifuged (1,500 × *g*, 10 min), and analyzed using the LDH kit according to the manufacturer's instructions. Absorbance at 490 nm was measured using a BioTek Synergy H1 microplate reader and normalized to the standard curve.

### 2.6 Cell cycle analysis by flow cytometry

The cell cycle was analyzed using a DCFH-DA fluorescence probe kit (Beyotime). Cells were stained with propidium iodide (PI). DNA content analysis was performed using a BD FACS Canto II flow cytometer, and cell cycle distribution was quantified using FlowJo (version 10.8.1).

### 2.7 Western blotting

The cells were collected using a cell scraper, washed twice with cold PBS, and lysed in RIPA buffer (Beyotime). Protein concentrations in the lysates were determined using the BCA assay. Proteins were separated by SDS-PAGE and transferred to PVDF membranes (Millipore, Billerica, United States). The membranes were then incubated with primary antibodies against NLRP3 (1:900), p-P65 (1:1,000), p-P38 (1:1,000), GAPDH (1:20,000), and IL-6 (1:1,000). Following incubation with secondary antibodies (ZSGB-BIO, Beijing, China), bands were detected using a Bio-Rad gel imaging system, and band intensities were analyzed with ImageJ software.

### 2.8 Wound healing assay

Cells were seeded into 6-well plates and cultured at full confluence (100%). A uniform linear wound was created in the monolayer using a sterile 200 μl pipette tip. After washing three times with PBS to remove dislodged cells, fresh serum-free medium was added. The plates were incubated under standard culture conditions (37 °C, 5% CO_2_) for 24 h. Wound closure dynamics were monitored at 0 and 24 h using an inverted phase-contrast microscope (Nikon, Japan). Quantitative analysis of migration was performed by measuring the denuded area at each time point with ImageJ software (v1.53, NIH, United States) with the “Wound Healing Size Tool” plugin. The wound healing rate was calculated as: Wound healing rate (%) = [(initial area–area at time) ÷ Initial area] × 100.

### 2.9 Capillary tube formation assay

Growth factor-reduced Matrigel (Corning #356231, Bedford, MA, United States) was polymerized in 96-well plates (50 μl/well) at 37 °C for 45 min. H9C2 cells (6 × 10^4^ cells/well) suspended in complete medium were seeded onto the Matrigel-coated wells under four experimental conditions: control, LDL, GPs, and LDL+GPs. After 4 h of incubation, tubular network formation was imaged using an inverted microscope ( × 40 magnification). Quantification of angiogenic-like behavior was performed using ImageJ with the “Angiogenesis Analyzer” plugin, evaluating total tube length and branch points per field.

### 2.10 Nitric oxide and myeloperoxidase (MPO) assays

Nitric oxide (NO) production was quantified using the Nitric Oxide Assay Kit (Jiancheng # A012-1-2, Nanjing, China). Briefly, 500 μl of cell supernatant was mixed with 600 μl of Griess reagent and incubated at 25 °C for 10 min. Absorbance was measured at 550 nm using a Beckman Coulter microplate reader.

MPO activity was determined using a commercial MPO Detection Kit (Nanjing Jiancheng Bioengineering Institute #A044-1-1) following the manufacturer's protocol. Supernatant samples (100 μl) were mixed with 2.9 ml of assay reagents, and the enzymatic reaction was monitored by measuring absorbance at 460 nm. MPO activity was calculated as follows: MPO activity (U/g tissue) = (A_sample–A_blank) ÷ (11.3 × W), where 11.3 is the formula coefficient and W is the sample weight (g), calculated as reagent volume (5 or 10%) × sample volume (0.18 ml).

### 2.11 RNA isolation and real-time quantitative PCR (RT-qPCR)

Total RNA was extracted using the RNA-easy Isolation Kit (Vazyme #RC112-01, Nanjing, China). cDNA synthesis was performed with PrimeScript™ RT Master Mix (Takara #RR036A, Kusatsu, Japan). RT-qPCR reactions were conducted using SYBR™ Green PCR Master Mix (Thermo Fisher #4309155, Waltham, MA, United States) on a QuantStudio 5 Real-Time PCR System (Applied Biosystems, Foster City, CA, United States). U6 snRNA served as the endogenous control for normalization of miR-223. Primer sequences were as follows:

miR-223-forward: 5′-GCGCGTGTCAGTTTGTCAAA-3′

miR-223-reverse: 5′-AGTGCAGGGTCCGAGGTATT-3′

U6-forward: 5′-CTCGCTTCGGCAGCACA-3′

U6-reverse: 5′-AACGCTTCACGAATTTGCGT-3′

## 3 Results

### 3.1 Elevated LDL-C as a critical risk factor for metabolic dysregulation

Our analysis of 19,862 participants revealed significant intergroup differences in gender distribution, age stratification, lipid profiles, glucose metabolism, and other metabolic parameters among the elevated LDL-C (ELC), normal LDL-C (NLC), and below-normal LDL-C (BLC) groups (Table 1). These differences provide a foundation for investigating LDL-C's role in metabolic dysregulation.

**Table 1:** Demographic and baseline characteristics of the study population.

Characteristics	Total (*N* = 19,862)	BLC group (*N* = 7,299)	NLC group (*N* = 8,295)	ELC group (*N* = 4,268)	χ^2^/*p*-value
Gender, *n* (%)					
Male	16,773 (84.45%)	5,824 (79.79%)	7,196 (86.75%)	3,753 (87.93%)	χ^2^ = 193.469/ *p* < 0.001
Female	3,089 (15.55%)	1,475 (20.21%)	1,099 (13.25%)	515 (12.07%)	
Age (years), *n* (%)					
< 20	183 (0.92%)	109 (1.49%)	54 (0.65%)	20 (0.47%)	χ^2^ = 570.504/ *p* < 0.001
20–29	8,932 (44.97%)	3,931 (53.86%)	3,408 (41.08%)	1,593 (37.32%)	
30–39	4,039 (20.34%)	1,414 (19.37%)	1,768 (21.31%)	857 (20.08%)	
40–49	2,535 (12.76%)	785 (10.75%)	1,140 (13.74%)	610 (14.29%)	
50–59	3,531 (17.78%)	869 (11.91%)	1,625 (19.59%)	1,037 (24.30%)	
>60	642 (3.23%)	191 (2.62%)	300 (3.62%)	151 (3.54%)	
WBCs, *n* (%)					
< 4 × 10^9^/L	467 (2.35%)	230 (3.15%)	161 (1.94%)	76 (1.78%)	χ^2^ = 54.677/ *p* < 0.001
4 × 10^9^/L−10 × 10^9^/L	18,723 (94.27%)	6,873 (94.16%)	7,844 (94.56%)	4,006 (93.86%)	
>10 × 10^9^/L	672 (3.38%)	196 (2.69%)	290 (3.50%)	186 (4.36%)	
Glucose, *n* (%)					
< 3.9 mmol/L	2,630 (13.24%)	1,050 (14.39%)	1,131 (13.63%)	449 (10.52%)	χ^2^ = 107.863/ *p* < 0.001
3.9–6.1 mmol/L	16,273 (81.93%)	5,999 (82.19%)	6,758 (81.47%)	3,516 (82.38%)	
>6.1 mmol/L	959 (4.83%)	250 (3.43%)	406 (4.89%)	303 (7.10%)	
Systolic blood pressure, *n* (%)					
< 90 mmHg	74 (0.37%)	42 (0.58%)	23 (0.28%)	9 (0.21%)	χ^2^ = 234.507/ *p* < 0.001
90–139 mmHg	16,341 (82.27%)	6,351 (87.01%)	6,700 (80.77%)	3,290 (77.09%)	
>139 mmHg	3,447 (17.35%)	906 (12.41%)	1,572 (18.95%)	969 (22.70%)	
BMI, *n* (%)					
< 18.5 kg/m^2^	853 (4.29%)	562 (7.70%)	230 (2.77%)	61 (1.43%)	χ^2^ = 1,058.098/ *p* < 0.001
18.5–23.9 kg/m^2^	9,164 (46.14%)	4,089 (56.02%)	3,563 (42.95%)	1,512 (35.43%)	
>23.9 kg/m^2^	9,845 (49.57%)	2,648 (36.28%)	4,502 (54.27%)	2,695 (63.14%)	

ELC, elevated LDL-C (ELC) group; NLC, normal LDL-C group; BLC, below LDL-C group.

The ELC group demonstrated distinct demographic characteristics, with males constituting 87.93% of the cohort, a significantly higher proportion than in the other groups. In contrast, the BLC group showed a female predominance, suggesting gender-specific patterns of LDL-C regulation. Age stratification revealed a predominance of middle-aged (50–59 years: 24.30%) and elderly (≥60 years: 3.54%) individuals in the ELC group (Table 1).

**Figure 1:**
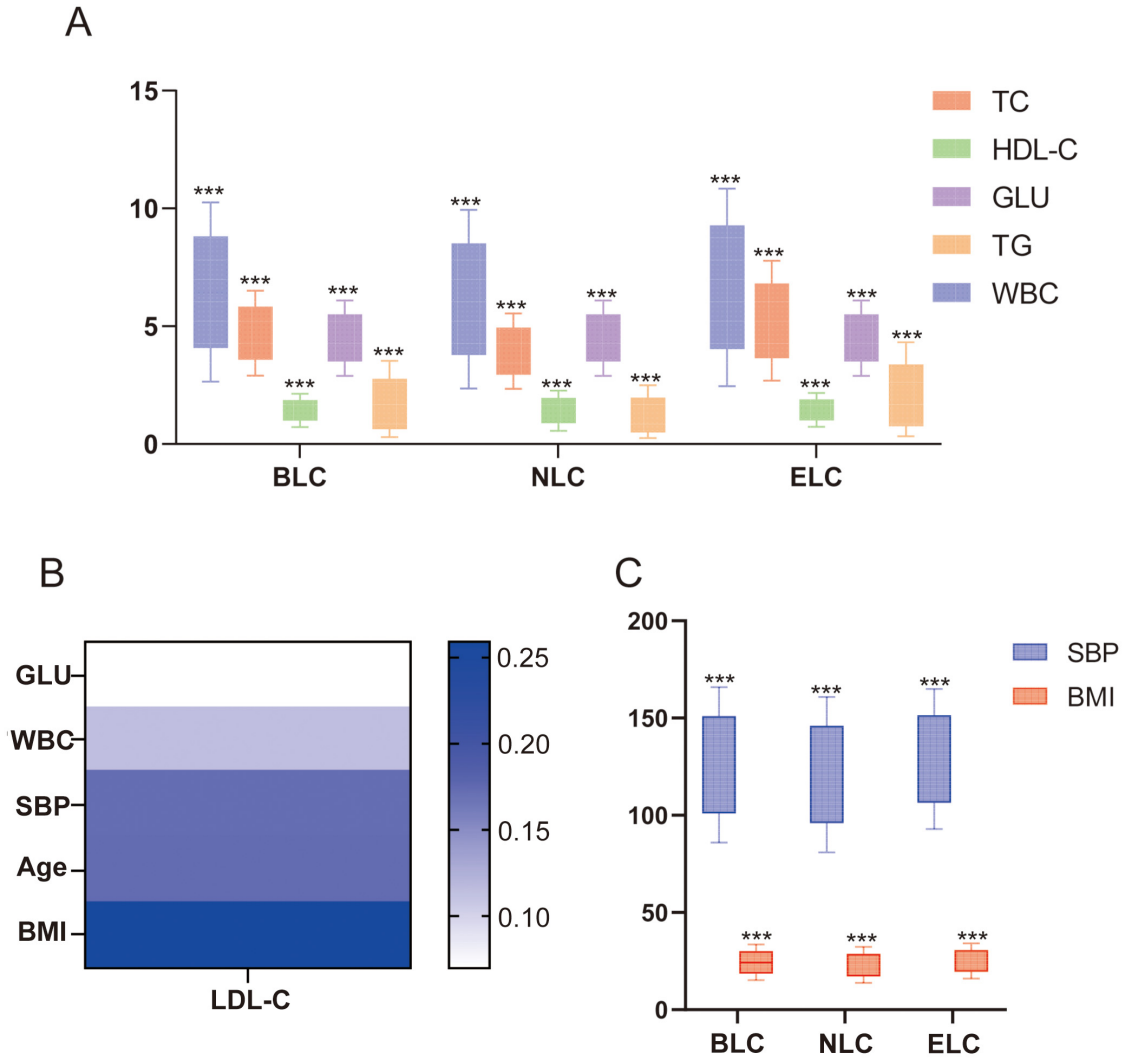
Population distributions of metabolic parameters. Cross-sectional analysis of adults undergoing routine health examinations. Total *N* = 19,862. **(A)** Distribution of TC, HDL-C, GLU, TG, and WBC across the BLC, NLC, and ELC groups. **(B)** Correlation analysis of LDL-C with GLU, WBC, SBP, age, and BMI. **(C)** Distribution of SBP and BMI across the BLC, NLC, and ELC groups. All data are presented as mean ± S.D. ***p < 0.001 between groups. The symbols indicate statistical significance, with horizontal lines showing the pairwise comparisons between groups. TC, total cholesterol; HDL-C, high-density lipoprotein cholesterol; LDL-C, low-density lipoprotein cholesterol; TG, triglycerides.

Notably, the ELC group exhibited elevated blood glucose (GLU) levels and leukocytosis (WBC >10 × 10^9^/L: 4.36%), the latter indicating potential systemic inflammation (Table 1). Lipid profiling demonstrated significantly higher total cholesterol (TC) and triglyceride (TG) levels, along with lower HDL-C levels, in the ELC group compared with the NLC and BLC groups (Figure 1A). Metabolic parameters showed greater BMI variability and elevated systolic blood pressure (SBP) in the ELC group (Figure 1C), consistent with known associations between dyslipidemia and vascular dysfunction. Correlation analysis (Figure 1B) revealed strong positive associations between LDL-C and BMI (*r* = 0.259), followed by moderate associations with SBP (*r* = 0.172) and WBCs (*r* = 0.116), suggesting multisystemic metabolic and inflammatory effects.

Multivariate regression (Table 2) confirmed significant associations between LDL-C and WBCs (β = 0.019, *p* < 0.001), BMI (β = 0.037, *p* < 0.001), and SBP (β = 0.042, *p* < 0.001). These findings establish elevated LDL-C as a systemic risk factor that influences both metabolic homeostasis and inflammatory pathways, potentially amplifying CVD risk.

**Table 2:** Multivariate linear regression analysis of factors influencing LDL-C levels.

Variables	Unstandardized coefficients (*B*)		Standardized coefficients (β)	*t*	*p*	Collinearity diagnostics	
	*B*	Standard error	β			VIF	Tolerance
Constant	−0.405	0.054	–	−7.449	< 0.001	–	–
Age	−0.001	0.000	−0.012	−1.997	0.046	1.325	0.755
WBCs	0.010	0.003	0.019	3.332	< 0.001	1.092	0.915
Blood glucose	−0.023	0.004	−0.032	−5.596	< 0.001	1.149	0.871
Blood pressure	0.002	0.000	0.042	6.832	< 0.001	1.303	0.768
BMI	0.009	0.002	0.037	5.666	< 0.001	1.454	0.688
Total cholesterol	0.705	0.007	0.701	103.069	< 0.001	1.606	0.623
Triglycerides	−0.053	0.004	−0.094	−14.908	< 0.001	1.391	0.719
HDL-C	−0.470	0.018	−0.162	−25.862	< 0.001	1.365	0.733
*R* ^2^	0.428						
Adjusted *R*^2^	0.427						
*F*	*F*_(8, 19853)_ = 1,853.669, *p* = 0.000						
Durbin–Watson (D–W)	0.684						

Dependent variable = low-density lipoprotein cholesterol (LDL-C).

### 3.2 GPs attenuate LDL-mediated cardiomyocyte dysfunction

Beyond its metabolic effects on LDL-C, LDL exerts direct cardiotoxicity through its multicomponent structure (cholesterol, lipids, and apolipoproteins). Using H9C2 cardiomyocytes exposed to physiologically relevant LDL concentrations (0.25 mg/ml, mildly reduced LDL level; 1.3 mg/ml, normal human physiological level; and 3 mg/ml, pathological hyperlipidemia threshold), we investigated LDL-induced cellular dysfunction and the protective effects of 0.5 mg/ml of GPs, a concentration previously established for cytoprotection.

**Figure 2:**
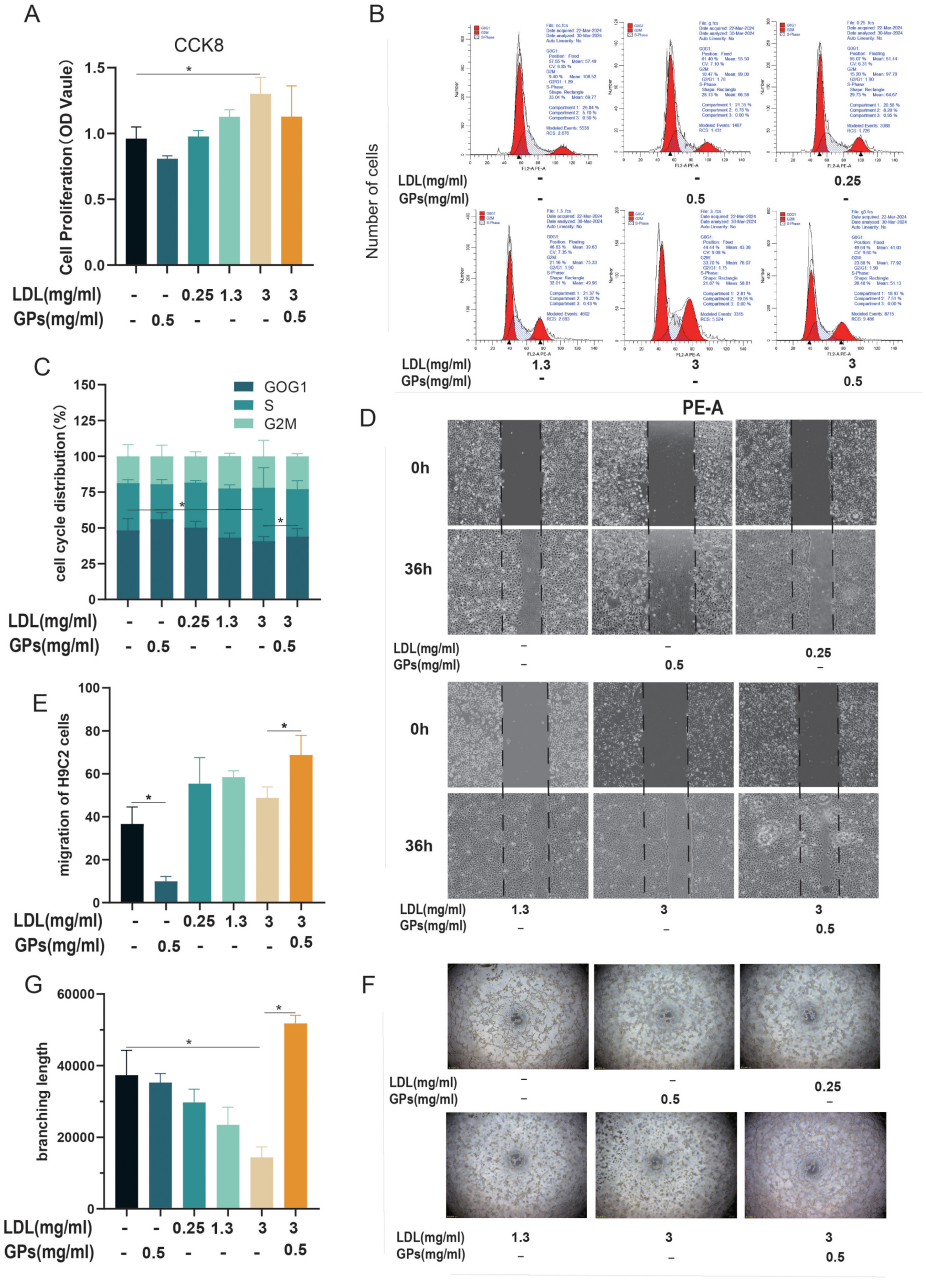
Proliferative and migratory responses of H9C2 cardiomyocytes following LDL and GPs exposure. H9C2 cells were exposed to LDL (0.25, 1.3, or 3 mg/ml) with or without GPs (0.5 mg/ml) under the conditions indicated below each panel; wound healing images were acquired at 0 and 36 h. **(A)** CCK-8 assay for cell viability (OD value at 450 nm). **(B)** Representative flow cytometry histograms of cell cycle distribution. **(C)** Quantification of cell cycle phase proportions (G0/G1, S, G2/M). **(D)** Representative wound healing images. **(E)** Quantification of cell migration in the wound healing assay. **(F)** Representative tube-formation images on Matrigel. **(G)** Quantification of total branching length. *N* = 3 independent samples per group. All data are presented as mean ± S.D. *p < 0.05 between groups. The symbols indicate statistical significance, with horizontal lines showing pairwise comparisons between groups. CCK-8, cell counting kit-8; OD, optical density; GPs, gypenosides; LDL, low-density lipoprotein.

Comprehensive functional analyses revealed concentration-dependent effects of LDL on cardiomyocyte pathophysiology (Figures 2A–G). CCK-8 assays demonstrated a paradoxical proliferative stimulation induced by LDL (peak effect at 3 mg/ml, *p* < 0.01 vs. control), which was significantly attenuated by GPs co-treatment (Figure 2A). Cell cycle profiling further elucidated this phenomenon, with high-dose LDL reducing G1-phase occupancy (indicating accelerated G1/S transition), whereas GPs monotherapy prolonged the G1 phase (Figure 2B). Notably, GPs supplementation partially normalized the cell cycle distribution in LDL-challenged cells (Figure 2C).

Functional impairment was evident in wound-healing assays, where LDL dose-dependently suppressed migratory capacity (62% reduction at 3 mg/ml, ^*^p < 0.001), an effect substantially rescued by GPs co-administration (Figures 2D, E). Assessment of angiogenic-like behavior using tube formation assays revealed similar patterns: 3 mg/ml of LDL decreased total vascular branch length by 45% (*p* < 0.01), while GPs combination treatment restored network complexity to near-baseline levels (Figures 2F, G). Intriguingly, GPs monotherapy showed negligible effects on angiogenesis, suggesting context-dependent therapeutic synergy.

### 3.3 Oxidative-inflammatory mechanisms underlying LDL-induced cardiotoxicity

To further elucidate the mechanisms underlying the detrimental effects of LDL on cardiomyocyte function, we systematically evaluated the effects of LDL and GPs on oxidative stress and inflammatory markers in H9C2 cardiomyocytes. Our findings revealed that LDL aggravated oxidative damage, whereas GPs exerted cytoprotective effects through dual modulation of redox homeostasis and inflammatory signaling pathways.

**Figure 3:**
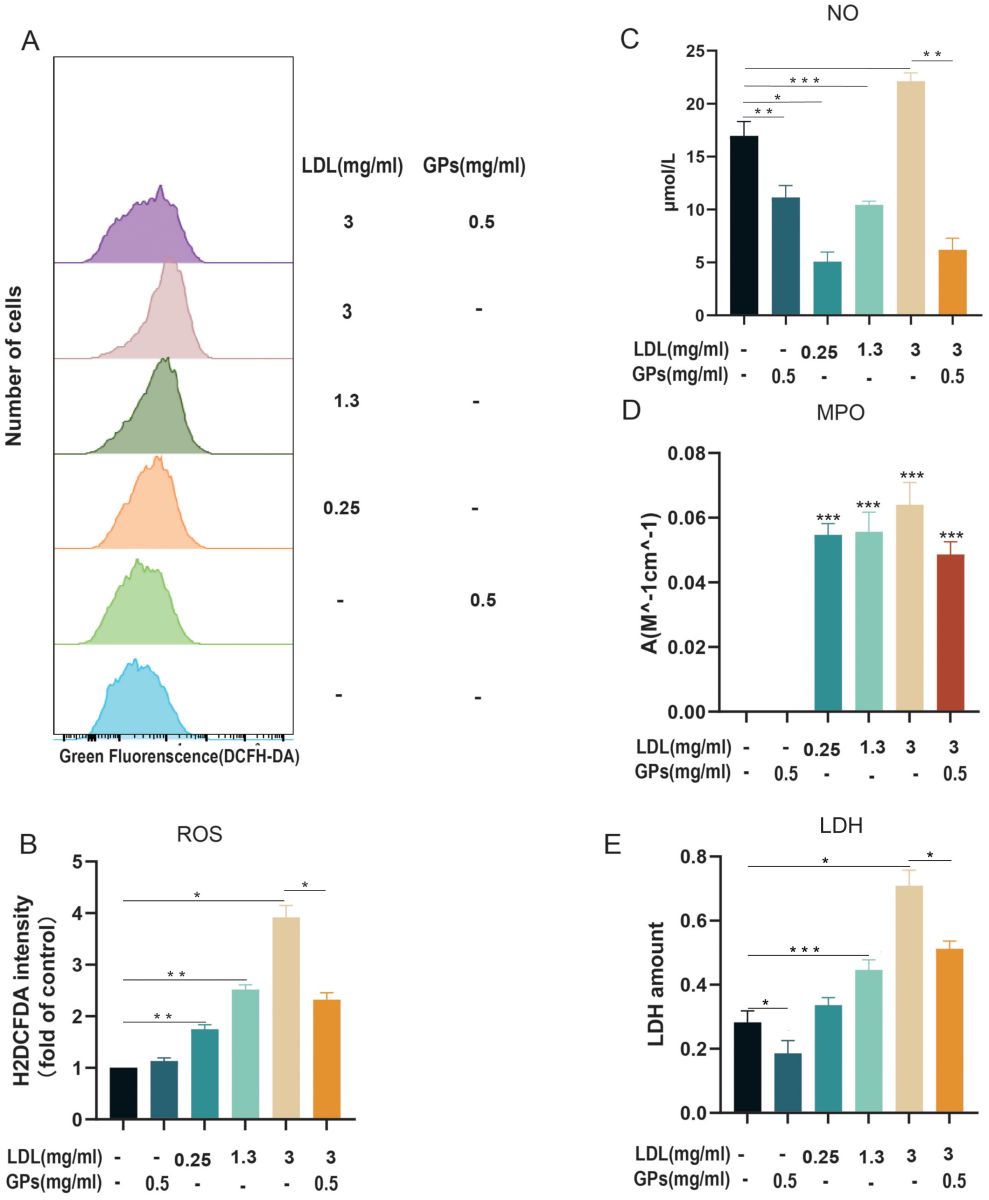
Oxidative stress and injury markers following LDL stimulation and GPs intervention in H9C2 cardiomyocytes. Cells were treated with LDL (0.25, 1.3, or 3 mg/ml) with or without GPs (0.5 mg/ml) as indicated. **(A)** Representative flow cytometry histograms of DCF fluorescence after DCFH-DA staining. **(B)** Quantification of intracellular ROS as H2DCFDA intensity (fold of control). **(C)** NO production. **(D)** MPO activity. **(E)** LDH release. *N* = 3 independent samples per group. All data are presented as mean ± S.D. *p < 0.05, **p < 0.01, and ***p < 0.001 between groups. The symbols indicate statistical significance, with horizontal lines showing pairwise comparisons between groups. DCFH-DA, 2′,7′-dichlorodihydrofluorescein diacetate; ROS, reactive oxygen species; NO, nitric oxide; MPO, myeloperoxidase; LDH, lactate dehydrogenase; LDL, low-density lipoprotein; GPs, gypenosides.

As shown in Figures 3A, B, LDL treatment induced a dose-dependent increase in intracellular ROS levels (*p* < 0.05), with maximal activation observed at 3 mg/ml of LDL. Conversely, GPs monotherapy significantly attenuated basal ROS generation. Notably, co-treatment with GPs effectively reversed LDL-induced ROS overproduction by 40.69% (vs. LDL alone).

For inflammatory response analysis, the pro-inflammatory effects of LDL were evidenced by elevated NO levels (Figure 3C), which increased by 2.1-fold at 3 mg/ml of LDL compared to controls (*p* < 0.05). GPs intervention suppressed NO synthesis to near-baseline levels in both the monotherapy group (34.29% reduction) and the combination group (63.49% reduction). Although MPO activity (Figure 3D) showed only a marginal elevation in LDL-treated cells, the significant reduction in MPO in the co-treatment group further substantiated the anti-inflammatory properties of GPs.

For cytoprotective efficacy, LDH leakage assays (Figure 3E) confirmed LDL-induced loss of integrity, with 3 mg/ml of LDL increasing LDH release (^*^p < 0.01). Remarkably, GPs co-administration reduced LDH efflux compared with LDL treatment alone, achieving levels comparable to those of untreated controls.

### 3.4 GPs-mediated modulation of the NLRP3/miR-223 signaling axis

To investigate the molecular mechanisms underlying LDL-induced inflammatory injury in H9C2 cardiomyocytes, we systematically analyzed activation of the NLRP3 inflammasome pathway and associated signaling molecules using Western blot and RT-qPCR.

**Figure 4:**
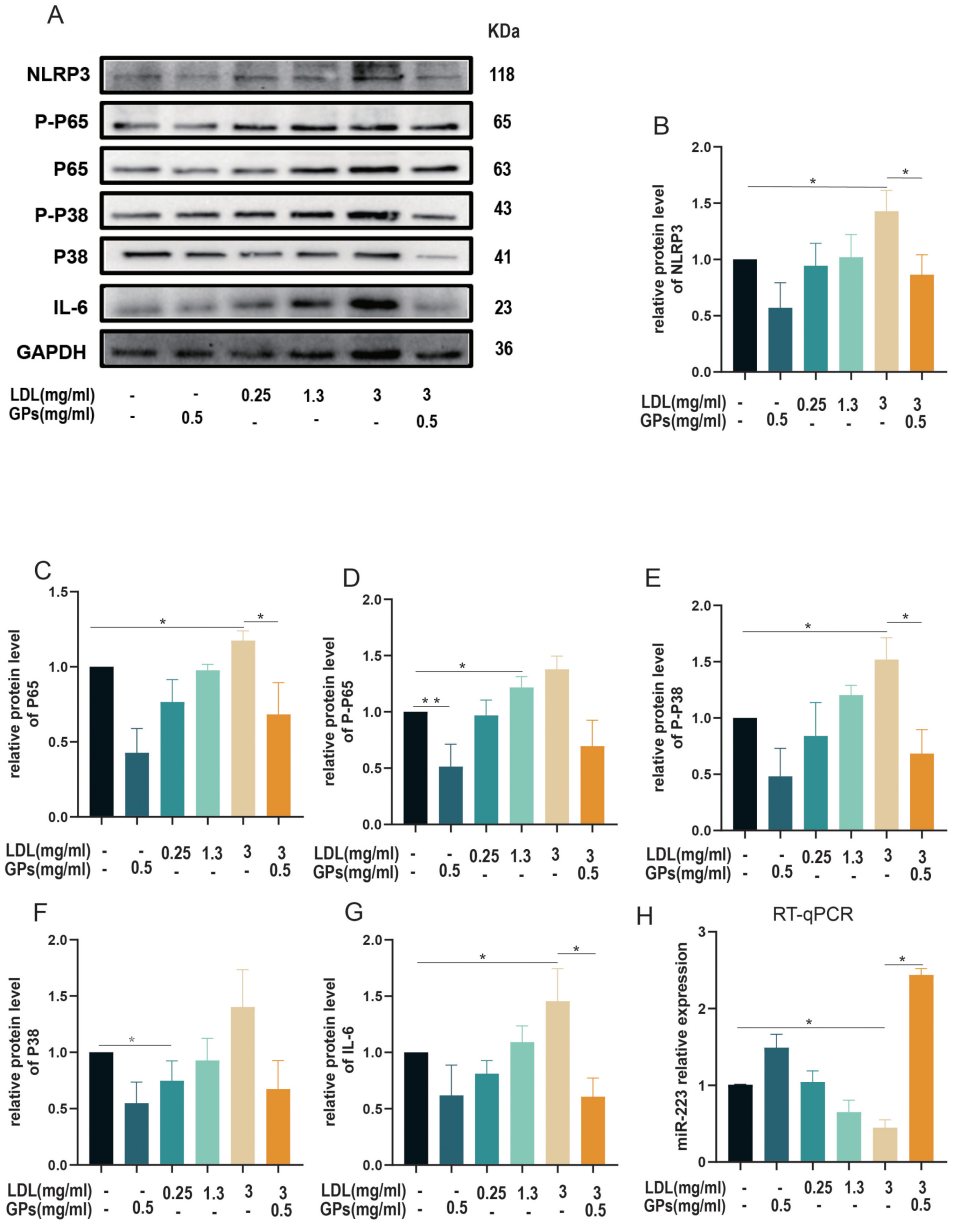
Dose-dependent regulation of the miR-223/NLRP3 inflammatory axis by LDL and GPs. H9C2 cardiomyocytes were treated with increasing concentrations of LDL (0.25, 1.3, or 3 mg/ml) with or without GPs (0.5 mg/ml) as indicated. **(A)** Representative immunoblots for NLRP3, P-P65, P65, P-P38, P38, IL-6, and GAPDH. **(B–G)** Densitometric quantification of the corresponding proteins normalized to GAPDH and expressed relative to the control group. **(H)** RT-qPCR quantification of miR-223 expression (normalized to U6 and expressed relative to control). Sample size: *n* = 3 independent samples per group. All data are presented as mean ± S.D. *p < 0.05, **p < 0.01 between groups. The symbols indicate statistical significance, with horizontal lines showing pairwise comparisons between groups. NLRP3, NOD-like receptor family pyrin domain containing 3; NF-κB, nuclear factor kappa B; IL-6, interleukin-6; GAPDH, glyceraldehyde-3-phosphate dehydrogenase; RT-qPCR, reverse transcription quantitative polymerase chain reaction; LDL, low-density lipoprotein; GPs, gypenosides.

Western blot analysis (Figures 4A–G) revealed a dose-dependent upregulation of NLRP3 (Figure 4B), p65 (Figure 4C), the p-p65/p65 ratio (Figure 4D), p38 (Figure 4E), the p-p38/p38 ratio (Figure 4F), and IL-6 (Figure 4G) expression in LDL-treated groups (*p* < 0.05). Maximum activation was observed at 3 mg/ml of LDL (*p* < 0.05), indicating substantial NLRP3 inflammasome activation and subsequent engagement of the NF-κB and MAPK signaling pathways. Conversely, treatment with GPs alone markedly downregulated these inflammatory mediators (*p* < 0.05). Co-treatment with GPs significantly attenuated the LDL-induced inflammatory response (*p* < 0.05 vs. LDL group).

RT-qPCR analysis of miR-223 expression (Figure 4H) showed significant suppression following LDL treatment (lowest at 3 mg/ml, *p* < 0.05), whereas GPs treatment significantly upregulated miR-223 compared with controls (*p* < 0.05). Notably, co-treatment restored miR-223 expression to levels comparable to those in the GPs-only group, suggesting miR-223-mediated regulation of NLRP3 inflammasome activity.

### 3.5 Functional validation of the miR-223/NLRP3 regulatory axis

After elucidating the mechanism by which GPs alleviate LDL-induced inflammatory damage in H9C2 cardiomyocytes, we further investigated the upstream regulatory pathways involved. Through combined Western blot and RT-qPCR analyses with miR-223 inhibition, we systematically examined the regulatory role of GPs in LDL-induced inflammation.

**Figure 5:**
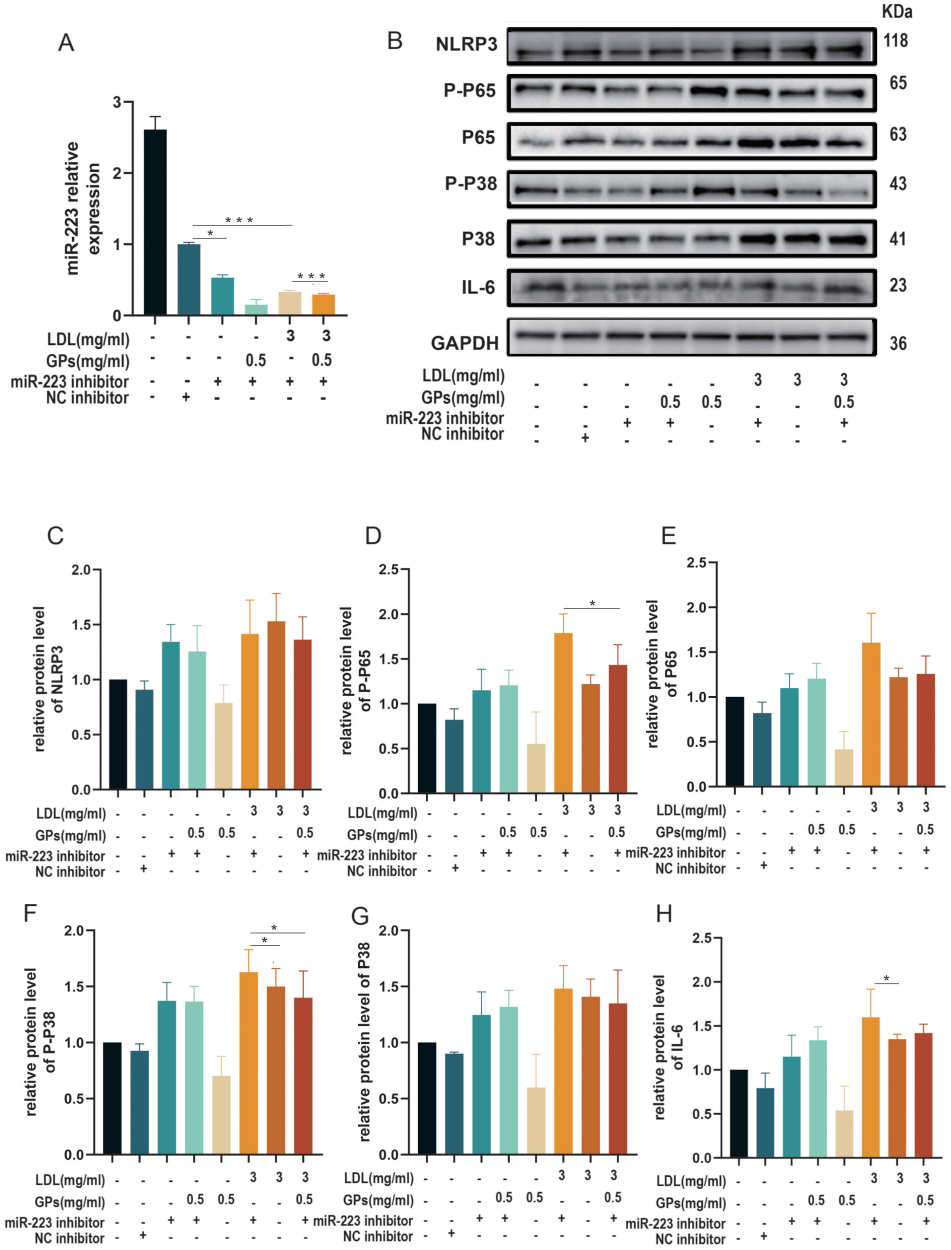
miR-223 mediates the anti-inflammatory effects of GPs in LDL-stimulated H9C2 cardiomyocytes. Cells were transfected with a miR-223 inhibitor or an NC inhibitor and then treated with LDL (3 mg/ml) and/or GPs (0.5 mg/ml) as indicated beneath each panel. **(A)** RT-qPCR measurement of miR-223 expression; values are presented relative to the NC inhibitor group (set to 1.0). **(B)** Representative immunoblots for NLRP3, P-P65, P65, P-P38, P38, IL-6, and GAPDH. **(C–H)** Densitometric quantification of NLRP3, P65, P-P65, P-P38, P38, and IL-6 normalized to GAPDH and expressed relative to the NC inhibitor group. *N* = 3 independent samples per group. All data are presented as mean ± S.D. *p < 0.05, ***p < 0.001 between groups. The symbols indicate statistical significance, with horizontal lines showing pairwise comparisons between groups. NC, negative control; NLRP3, NOD-like receptor family pyrin domain containing 3; NF-κB, nuclear factor kappa B; IL-6, interleukin-6; GAPDH, glyceraldehyde-3-phosphate dehydrogenase; RT-qPCR, reverse transcription quantitative polymerase chain reaction; LDL, low-density lipoprotein; GPs, gypenosides.

The RT-qPCR results (Figure 5A) demonstrated successful miR-223 knockdown, with expression levels in the inhibitor group reduced by 50% compared to controls (*p* < 0.05). Notably, although the miR-223 level in the NC inhibitor group was mildly reduced compared with the untreated control, this reduction did not coincide with coordinated activation of the NLRP3/NF-κB/p38/IL-6 axis (Figures 5B–H), supporting the specificity of the miR-223 inhibitor. miR-223 expression was significantly reduced in the GPs + miR-223 inhibitor group compared with other treatment conditions. In LDL-stimulated cells, GPs treatment increased miR-223 expression, whereas miR-223 inhibition markedly suppressed this restoration.

Western blot analyses (Figures 5B–H) revealed significant upregulation of NLRP3-associated inflammatory signaling in miR-223-inhibited cells. Specifically, NLRP3 expression increased 1.48-fold (Figure 5C), while the phosphorylation ratios of p65 (p-p65/p65; Figure 5D) and p38 MAPK (p-p38/p38; Figure 5F) in the LDL (3 mg/ml) + miR-223 inhibitor group increased by 11.47 and 9.92%, respectively (all *p* < 0.05). Corresponding increases in total p65 (Figure 5E), p38 (Figure 5G), and IL-6 (Figure 5H) levels further supported activation of downstream inflammatory pathways.

Crucially, miR-223 knockdown attenuated the anti-inflammatory efficacy of GPs. The combination group exhibited higher expression of NLRP3 (Figure 5C), p-p65 (Figure 5D), p65 (Figure 5E), p-p38 (Figure 5F), p38 (Figure 5G), and IL-6 (Figure 5H) compared with GPs-treated controls (*p* < 0.05). GPs treatment alleviated LDL-induced inflammatory changes, whereas miR-223 inhibition largely abolished the anti-inflammatory effects of GPs. This contrasted sharply with LDL-treated groups, where GPs effectively suppressed all measured inflammatory markers. These findings indicate that GPs primarily exert their protective effects through miR-223-mediated regulation of the NLRP3 pathway.

### 3.6 NLRP3 as the pivotal inflammatory mediator in LDL-induced cardiomyocyte injury

**Figure 6:**
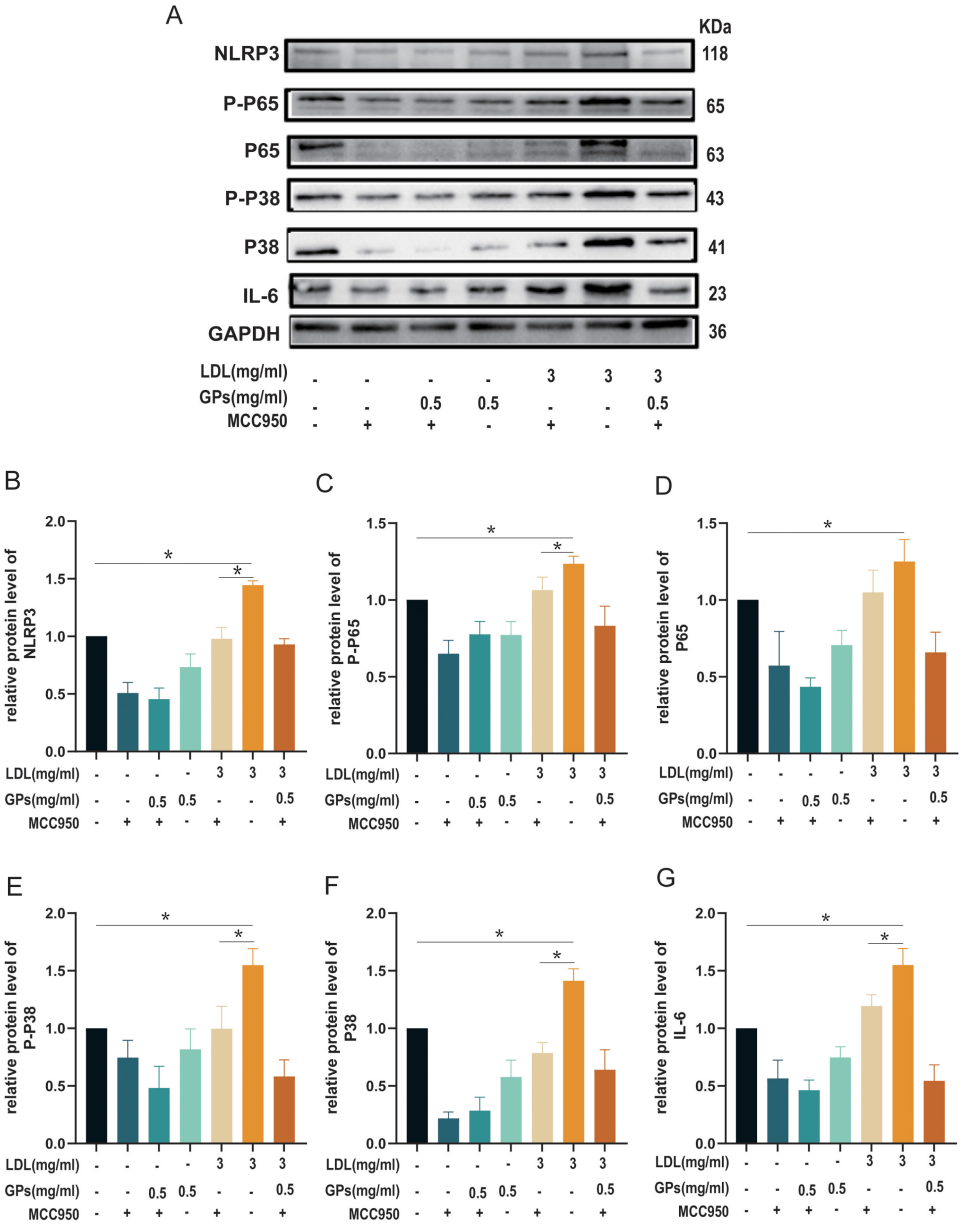
Pharmacological inhibition of NLRP3 modulates LDL-induced inflammatory signaling and the response to GPs. H9C2 cardiomyocytes were treated with LDL (3 mg/ml) and/or GPs (0.5 mg/ml) in the presence or absence of MCC950 (NLRP3 inhibitor; 10 μM) as indicated. **(A)** Representative immunoblots for NLRP3, P-P65, P65, P-P38, P38, IL-6, and GAPDH. **(B–G)** Densitometric quantification of NLRP3, P-P65, P65, P-P38, P38, and IL-6 normalized to GAPDH and expressed relative to the control group. *N* = 3 independent samples per group. All data are presented as mean ± S.D. *p < 0.05 between groups. The symbols indicate statistical significance, with horizontal lines showing pairwise comparisons between groups. MCC950, a selective NLRP3 inflammasome inhibitor; NLRP3, NOD-like receptor family pyrin domain containing 3; NF-κB, nuclear factor kappa B; IL-6, interleukin-6; GAPDH, glyceraldehyde-3-phosphate dehydrogenase; LDL, low-density lipoprotein; GPs, gypenosides.

To further elucidate the mechanistic relevance of the miR-223/NLRP3 axis, we systematically examined the effects of pharmacological NLRP3 inhibition using MCC950 on LDL-induced inflammatory signaling and the cardioprotective actions of GPs. These experiments provided additional evidence supporting the involvement of NLRP3-associated inflammatory signaling in LDL-mediated cellular responses and suggested that the protective effects of GPs are dependent on NLRP3-related regulatory mechanisms. Western blot analysis (Figures 6A–G) demonstrated that LDL exposure (3 mg/ml) significantly upregulated key components of the NLRP3 inflammasome pathway (all *p* < 0.05 vs. control), including NLRP3 protein expression (Figure 6B), NF-κB signaling markers (p-p65/p65 ratio in Figure 6C; total p65 in Figure 6D), p38 MAPK activation (p-p38/p38 ratio in Figure 6E; total p38 in Figure 6F), and pro-inflammatory cytokine IL-6 production (Figure 6G).

Notably, these LDL-induced molecular alterations (all *p* < 0.05 vs. control) were markedly attenuated by GPs treatment, demonstrating its potent anti-inflammatory properties.

## 4 Discussion

The global prevalence of hyperlipidemia has shown a persistent upward trend, particularly in urbanized regions and populations. Although hyperlipidemia often progresses asymptomatically in its early stages, emerging evidence suggests that subclinical impairment of cardiac structure and function may already occur during this latent period. This insidious nature underscores hyperlipidemia as a critical public health challenge worldwide (28–30). Our analysis of 19,862 participants revealed a robust association between elevated LDL-C levels and hyperlipidemia risk, with pronounced susceptibility among middle-aged and elderly men. Notably, males constituted 87.93% of the elevated LDL-C (ELC) cohort, with age stratification showing a prevalence of 24.30% in the 50–59 age group and 3.54% among those aged >60 years. These demographic patterns suggest significant age- and sex-dependent variations in LDL-C metabolism, corroborating previous reports of age-related LDL-C accumulation and consequent hyperlipidemia risk (3, 31). Future studies should use longitudinal designs and randomized controlled trials (RCTs) to elucidate the causal role of elevated LDL-C in the development of hyperlipidemia. It is also essential to control for potential confounders, including dietary factors, genetic predisposition, and environmental exposures, and to expand the study population to enhance the generalizability of the findings.

Intriguingly, our data demonstrated an inverse relationship in younger populations, with 53.86% of 20–29-year-olds falling into the below-normal LDL-C (BLC) category. This contrasts with studies documenting early-onset dyslipidemia, such as Lartey's report of 9.2% LDL-C elevation in Ghanaian children (31) and Raitakari's identification of adolescence as a critical risk period (32). Our findings emphasize the middle-aged (50–59 years) and older populations (>60 years) as priority targets for intervention. Mechanistically, LDL-C exerts systemic effects through metabolic dysregulation and inflammatory pathways, influencing blood glucose homeostasis and leukocyte profiles (32). Notably, LDL particles (containing cholesterol, lipids, and apolipoproteins) better reflect cardiovascular risk than isolated LDL-C measurements, as evidenced by their association with γ-glutamyl transferase (GGT) elevation and amplified cardiovascular mortality in individuals >65 years (16, 33). Collectively, our findings demonstrate that LDL overload induces cardiomyocyte dysfunction through multiple pathways, including aberrant proliferative activation, cell-cycle dysregulation, impaired migratory capacity, and angiogenesis-like behavior.

The NLRP3 inflammasome's established role in cardiovascular pathogenesis (34, 35) led us to investigate its involvement in LDL-mediated cardiotoxicity. Our experimental models demonstrated that LDL particles downregulate miR-223 expression, upregulate NLRP3-related inflammatory signaling, and activate p38 MAPK/NF-κB signaling cascades. These molecular alterations promoted pro-inflammatory cytokine release, disrupted cardiomyocyte proliferation/migration, and impaired angiogenic-like processes. Furthermore, LDL exposure induced oxidative stress via dual mechanisms: MPO-mediated hypochlorous acid generation and ROS/NO interaction leading to the formation of cytotoxic peroxynitrite (ONOO^−^). This oxidative-inflammatory crosstalk established a self-perpetuating cycle of myocardial damage, potentially explaining LDL's central role in hyperlipidemia-associated cardiovascular pathology (35). Crucially, NLRP3 inhibition with MCC950 reversed LDL-induced cardiomyocyte injury, positioning NLRP3 as both a mediator and a therapeutic target, rather than a mere bystander.

Current hyperlipidemia management relies on statins (36–38) and emerging PCSK9 inhibitors (39–41), yet limitations persist, including resistance, side effects, and cost barriers (42–44). This therapeutic gap has spurred interest in natural compounds with pleiotropic effects. Gypenosides (GPs) from *Gynostemma pentaphyllum* exhibit multimodal cardioprotection through NF-κB inhibition (45) and mitochondrial enhancement (46). In the present study, GPs also exhibit multimodal cardioprotection by modulating the miR-223/NLRP3 axis. Therefore, GPs may serve as a potential therapeutic agent to counteract LDL-induced myocardial injury in hyperlipidemia, given their promising multimodal cardioprotective effects.

Our study reveals a dual pathological mechanism by which LDL promotes cardiomyocyte dysfunction through two major pathways: first, LDL induces oxidative damage via ROS-mediated cascade reactions; second, LDL exacerbates cardiac damage via NO-driven inflammatory activation. GPs counteract these harmful effects through various mechanisms, namely, scavenging free radicals (reducing ROS), inhibiting inflammatory mediators (suppressing NO), and maintaining membrane stability (normalizing LDH). Further research indicates that LDL activates NLRP3-associated inflammatory signaling by coordinating NF-κB and p38 MAPK signaling pathways, while GPs exert cardioprotective effects by upregulating miR-223 and subsequently inhibiting the NLRP3-mediated inflammatory cascade.

One major limitation of the current study is the lack of further validation regarding the potential role of NLRP3-independent pathways in the anti-inflammatory effects of GPs. While this study focused on the miR-223-mediated NLRP3 inhibition, future research should incorporate a more comprehensive factorial experimental design, such as an LDL+GPs group without MCC950, to further explore the NLRP3-independent mechanisms of GPs. Such additional studies will provide a more thorough understanding of the role of GPs in LDL-induced cardiomyocyte injury. Furthermore, although the miR-223/NLRP3 axis has been established as a key regulatory mechanism, validation of downstream miR-223 target genes remains a crucial step in refining the causal relationships within this axis. This validation will provide stronger theoretical support for GPs as a potential therapeutic agent for hyperlipidemia-associated cardiovascular damage and lay a foundation for the clinical application of miR-223-targeted interventions.

While the use of H9C2 cells offers valuable preliminary insights, *in vivo* validation using rat models will be essential to confirm the findings and assess the relevance of these results in a physiological setting. Future research should focus on *in vivo* experiments, optimizing GPs' bioavailability, assessing their long-term efficacy, and evaluating their preventive effects across different CVD spectra. These studies will provide stronger experimental evidence for GPs as a potential therapeutic agent for hyperlipidemia-associated cardiovascular damage.

## 5 Conclusion

Our population-based analysis established that elevated LDL-C is a key driver of hyperlipidemia, particularly in aging males. Cellular studies identify NLRP3 as the central node in LDL-induced cardiotoxicity and demonstrate the therapeutic capacity of GPs through miR-223/NLRP3 modulation. These findings provide mechanistic justification for exploring GPs-based interventions against hyperlipidemia-associated cardiovascular injury.

## Data Availability

The original contributions presented in the study are included in the article/supplementary material, further inquiries can be directed to the corresponding authors.
